# Leopard subspecies conservation under climate and land‐use change

**DOI:** 10.1002/ece3.11391

**Published:** 2024-05-21

**Authors:** Charlotte Mitchell, Jamie Bolam, Laura D. Bertola, Vincent N. Naude, Lucas Gonçalves da Silva, Orly Razgour

**Affiliations:** ^1^ Biosciences University of Exeter Exeter UK; ^2^ Department of Biology University of Copenhagen Copenhagen Denmark; ^3^ Department of Conservation Ecology and Entomology Stellenbosch University Matieland South Africa; ^4^ Center for Sustainable Development University of Brasília Brasília Brazil

**Keywords:** climate change, gap analysis, intraspecific variability, *Panthera pardus*, protected areas, species distribution models

## Abstract

Predicting the effects of global environmental changes on species distribution is a top conservation priority, particularly for large carnivores, that contribute to regulating and maintaining ecosystems. As the most widespread and adaptable large felid, ranging across Africa and Asia, leopards are crucial to many ecosystems as both keystone and umbrella species, yet they are threatened across their ranges. We used intraspecific species distribution models (SDMs) to predict changes in range suitability for leopards under future climate and land‐use change and identify conservation gaps and opportunities. We generated intraspecific SDMs for the three western leopard subspecies, the African, *Panthera pardus pardus*; Arabian, *Panthera pardus nimr*; and Persian, *Panthera pardus tulliana*, leopards, and overlapped predictions with protected areas (PAs) coverage. We show that leopard subspecies differ in their environmental associations and vulnerability to future changes. The African and Arabian leopards are predicted to lose ~25% and ~14% of their currently suitable range, respectively, while the Persian leopard is predicted to experience ~12% range gains. We found that most areas predicted to be suitable were not protected, with only 4%–16% of the subspecies' ranges falling inside PAs, and that these proportions will decrease in the future. The highly variable responses we found between leopard subspecies highlight the importance of considering intraspecific variation when modelling vulnerability to climate and land‐use changes. The predicted decrease in proportion of suitable ranges falling inside PAs threatens global capacity to effectively conserve leopards because survival rates are substantially lower outside PAs due to persecution. Hence, it is important to work with local communities to address negative human‐wildlife interactions and to restore habitats to retain landscape connectivity where PA coverage is low. On the other hand, the predicted increase in range suitability across southern Europe presents opportunities for expansion outside of their contemporary range, capitalising on European rewilding schemes.

## Graphical abstract

This study uses intraspecific species distribution models to predict changes in range suitability for leopard subspecies under future climate and land‐use changes and to identify conservation gaps and opportunities through overlapping predictions with protected areas coverage. We focus on an iconic large carnivore of conservation concern, demonstrating the importance of using an intraspecific perspective when studying the impacts of global environmental changes on widespread taxa. We predict future range losses for some subspecies and a decline in the proportion of future suitable range falling within protected areas, threatening global capacity to effectively conserve leopards.

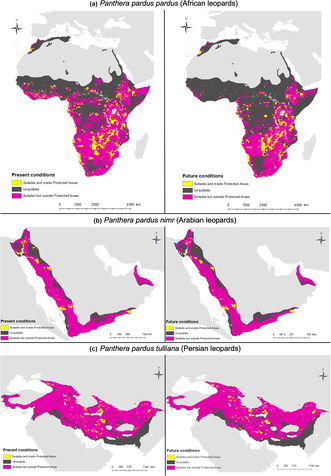

## 1 INTRODUCTION

Climate change is a major threat to biodiversity, which interacts with ongoing anthropogenic land‐use change and its associated risks (IPCC, 2022). Species are already shifting their distributions to track suitable conditions (Parmesan & Yohe, 2003) and range shifts are projected to accelerate in the future (Pecl et al., 2017). Species unable to move away from or adapt to these changes risk extinction (Araújo et al., 2019). Hence, predicting the effects of these global environmental changes on species distribution is a top conservation priority (Thuiller et al., 2008).

Apex predators have significant roles in maintaining ecosystems, supporting ecosystem health and influencing lower trophic levels (Atkins et al., 2019; Tshabalala et al., 2021). Their decline or extirpation often causes ecosystem‐wide biodiversity and species richness declines (Hollings et al., 2014). Despite their ecological importance, large terrestrial carnivores have experienced steep declines in both population size and geographic range over the past century, needing urgent conservation intervention (Abade et al., 2014). Large carnivore populations are primarily threatened by habitat loss and fragmentation, exacerbated by prey depletion and persecution (Ripple et al., 2014). These species are particularly vulnerable due to their small population sizes, high energy requirements, slow reproductive rates and wide roaming behaviour, which brings them into conflict with domestic livestock and humans (Cardillo et al., 2004; Ripple et al., 2014; Wolf & Ripple, 2016).

Leopards, *Panthera pardus*, are the most widespread and adaptable large felid, ranging across most of Africa and Asia, inhabiting various biomes, from tropical forests and savannas to alpine habitats and deserts (Jacobson et al., 2016). They can traverse and survive in highly transformed anthropogenic landscapes, including agricultural lands and urban fringes (Athreya et al., 2016; Braczkowski et al., 2018; Stein et al., 2011), where many are killed in retaliation to their real or perceived threat to livestock (Al‐Johany, 2007; Ebrahimi et al., 2017; Naude et al., 2020). Leopards have one of the broadest diets among carnivores (Hayward et al., 2006), feeding opportunistically on insects, reptiles, birds, small mammals and larger ungulates, depending on prey availability and pressure from competitors (Al‐Johany, 2007; Sari, 2022; Uphyrkina et al., 2001). Understanding future leopard distribution under predicted climate change is key to developing effective conservation strategies (Farashi & Shariati, 2018) in an increasingly human‐dominated landscape (Di Minin et al., 2016).

While behavioural plasticity allows leopards to persist where other big cats often cannot, this adaptability and wide geographic distribution has not protected them against the multitude of threats they face, having suffered global range declines of 63%–75%, exceeding the average of 53% large carnivore range loss (Jacobson et al., 2016). Leopards are classified as Vulnerable by the International Union for Conservation of Nature (IUCN; Stein, 2020) due to habitat loss, fragmentation, prey depletion, conflict with humans, unsustainable trophy hunting, poaching for body parts and indiscriminate killing (Jacobson et al., 2016). However, the status of the nine recognised subspecies ranges from Critically Endangered to Near Threatened (Stein, 2020). Leopards now occupy 25%–37% of their historic range, but 97% of this is occupied by the African (*P. p. pardus*), Indian (*P. p. fusca*) and Persian (*P. p. tulliana*) leopard subspecies, while Arabian (*P. p. nimr*) and Amur (*P. p. orientalis*) leopards have lost up to 98% of their former range (Jacobson et al., 2016) and remaining suitable habitats are predicted to decrease further (Zeng et al., 2022). Climate change poses a growing threat to leopards because its impacts on vegetation cover and prey availability will likely translate into individual fitness costs (Ebrahimi et al., 2017; Zeng et al., 2022). These impacts are likely to be exacerbated by changes to human distribution and activities due to climate change, which can further affect prey and habitat availability for leopards.

As highly ubiquitous and free‐roaming top carnivores (Tshabalala et al., 2021), leopards are crucial to many ecosystems as both keystone and umbrella species (Atkins et al., 2019; Hebblewhite et al., 2011). While leopard survival rates are often significantly higher in protected areas (PAs; Swanepoel et al., 2015; Thorn et al., 2012), such areas constitute only 17% of their remaining range (Jacobson et al., 2016). Thus, understanding how leopards respond to predicted climate and land‐use change is crucial to their conservation management and policy development (Asongu, 2013; Stein, 2020).

Species distribution models (SDMs), also referred to as ecological niche models when modelling species' environmental suitability (Peterson & Soberón, 2012), are one of the most common classes of biodiversity modelling, used to understand factors underpinning ecological patterns and forecast changes in potential species distributions under climate and land‐use changes (Araújo et al., 2019). SDMs are commonly applied in studies of biogeography, conservation biology, ecology, palaeoecology and wildlife management (Araújo & Guisan, 2006), across terrestrial, freshwater and marine environments and across spatial and temporal scales (Elith & Leathwick, 2009). SDMs can help inform long‐term conservation action by predicting potential future suitable areas and possible loss of present habitats (Schwartz, 2012). However, for species that are composed of separate subspecies or distinct evolutionary lineages, models generated for the species as a whole ignore local environmental adaptations and assume that current distributions reflect the entire set of suitable conditions (Razgour et al., 2019; Smith et al., 2019). Hence, models developed for individual subspecies or lineages can be more informative and produce more reliable and accurate predictions of change with meaningful conservation implications (Gonzalez et al., 2011).

In this study, we use SDMs to predict changes in range suitability for leopard subspecies under future climate and land‐use change to identify putative conservation gaps and opportunities. We generated intraspecific models for three leopard subspecies, the African, *Panthera pardus pardus*; Persian, *Panthera pardus tulliana*; and Arabian, *Panthera pardus nimr*, leopards. By overlapping predictions with protected areas coverage, this study aims to identify future changes in the proportion of protected potential suitable leopard range.

## 2 METHODS

### 2.1 Study species

African leopards are considered to have given rise to eight Middle Eastern (i.e., Arabian and Persian) and Asian (Indian; Sri Lankan, *P. p. kotiya*; Indochinese, *P. p. delacouri*; North‐Chinese, *P. p. japonensis*; Amur; and Javan, *P. p. melas*) leopard subspecies around 500–600 thousand years ago (Paijmans et al., 2021). African leopards once occurred across most of the African continent apart from the hyper‐arid interiors of the Sahara and Namib deserts but are now virtually extinct in North Africa, extremely rare throughout the West African coastal belt, and continue to decline outside of PAs across much of East and southern Africa, with only 33% of their historic habitat remaining (Jacobson et al., 2016). Leopard populations outside of Africa have fared little better, with the Arabian subspecies being limited to an estimated 100–250 individuals distributed across the remaining 2% of their habitat in the Middle Eastern states of Yemen, Oman and possibly the United Arab Emirates (Al‐Johany, 2007; Jacobson et al., 2016). No Arabian leopards remain in Saudi Arabia (Dunford et al., 2024). Leopard numbers have also experienced a significant reduction outside national parks across South‐East Asia with limited suitable habitat remaining across their historic range (Persian: 16%, Indian: 28%, Sri Lankan: 37%, Amur: 2%, Chinese: 2%, Indochinese: 4% and Javan: 16% (Jacobson et al., 2016)). In addition to the direct threat of vastly reduced habitat across their range, remaining leopard populations are becoming increasingly isolated by habitat fragmentation and the loss of connectivity. Here we modelled environmental suitability and then derived potential distribution for the three western leopard subspecies, African, Arabian and Persian, using the SDM algorithm Maxent (Phillips et al., 2006).

### 2.2 Location records

**FIGURE 1:**
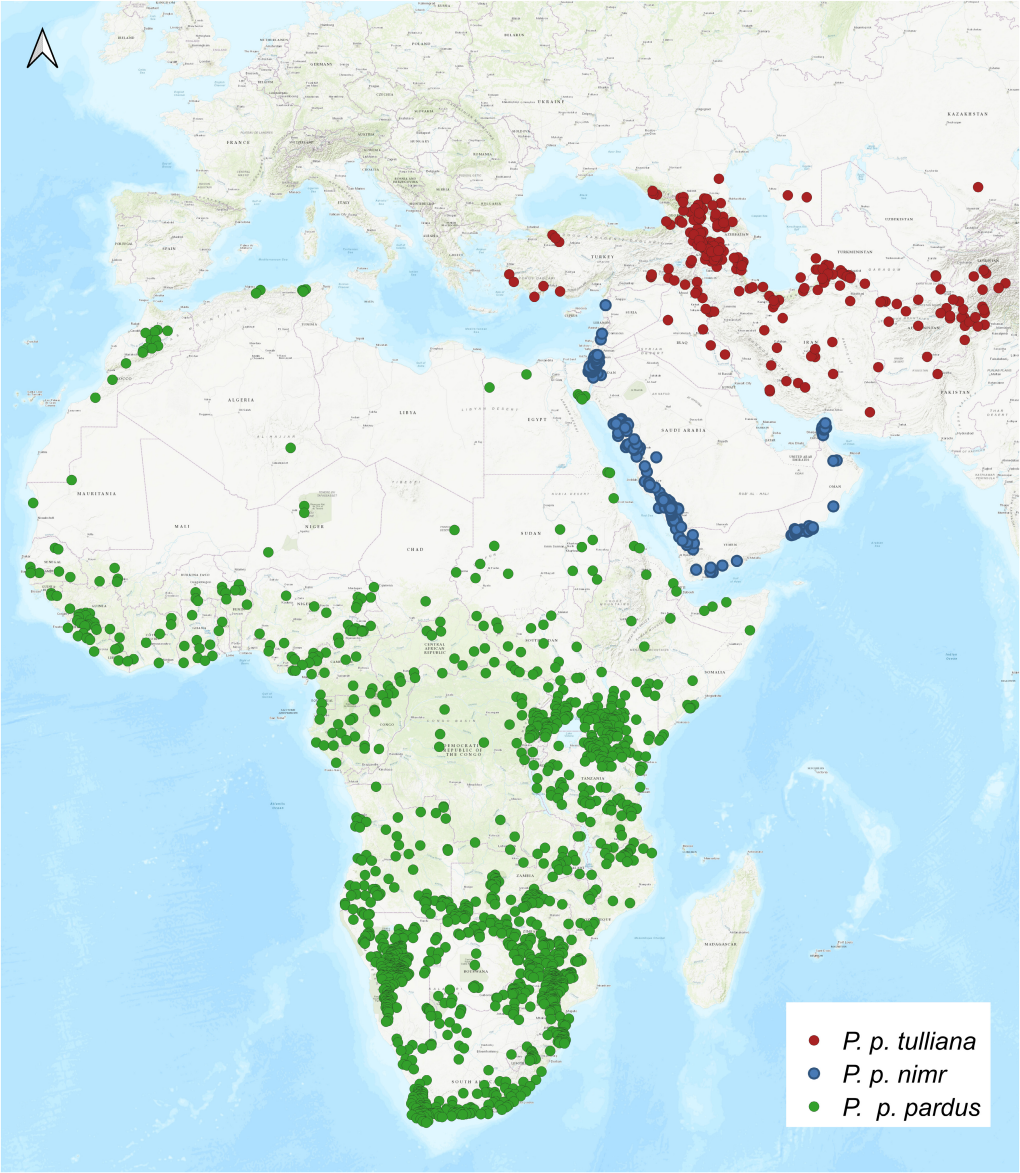
Current distributions of location records of the three western leopard subspecies included in this study (blue – Arabian leopard; green – African leopard; red – Persian leopard). Background map: ESRI World Topo.

Models were fitted with location records from 1970 onwards obtained from the online database GBIF (www.gbif.org/). To reduce spatial biases associated with under‐sampled areas and variation in data sharing (Beck et al., 2014), we also searched the scientific and grey literature to obtain additional location records from under‐represented areas (Data S2 for literature sources) and obtained unpublished location records from researchers. However, it is difficult to completely avoid all spatial bias, as bias is also apparent in research efforts. Visser et al. (2023) highlight this issue in African lions and the same is likely true for leopards. To correct for uneven sampling and clustering, records were thinned using the R package spThin (Aiello‐Lammens et al., 2015) to a distance of 10 km. We retained 1653 location records, 1271 for African, 163 for Arabian and 219 for Persian leopards (Figure 1; Dataset S1).

### 2.3 Environmental variables

Selection of environmental layers to include in the models was based on the published ecological requirements of the leopard subspecies and availability of future projections. Our SDMs included a combination of climatic (downloaded for 1981–2010 and 2041–2060 from Chelsa‐climate, https://chelsa‐climate.org/, at 30 arc sec, ~1 km resolution), land cover (Globio4 land cover map for 2015 and 2050; Schipper et al. (2020); at 10 arc sec, ~300 m resolution) and topographic (Worldclim, https://www.worldclim.org/, at 30 arc sec, ~1 km resolution) variables (Table S1). We used the General Circulation Model GFDL‐ESM4 with the more severe climate change scenario, ssp585. We reclassified the land cover map to 10 main categories relevant for leopards (Table S1). We used the R package raster (Hijmans, 2023) to test for collinearity among environmental variables, using Pearson correlations, and removed highly correlated variables (*r* > |.75|), retaining the variable with stronger contribution to model gain. The final models included 15 variables for the African leopard, 14 variables for the Arabian leopard and 13 variables for the Persian leopard.

Model resolution was set to 10 km to reflect the vast‐ranging behaviour of leopards. Study extent varied between the three subspecies to reflect their present distribution and potential future extent of suitable conditions. The African leopard model spanned Africa, Madagascar, most of Asia and Europe up to a latitude of 60° N. Madagascar was included as a theoretical exercise to investigate whether conditions are already or will become suitable there.

**FIGURE 2:**
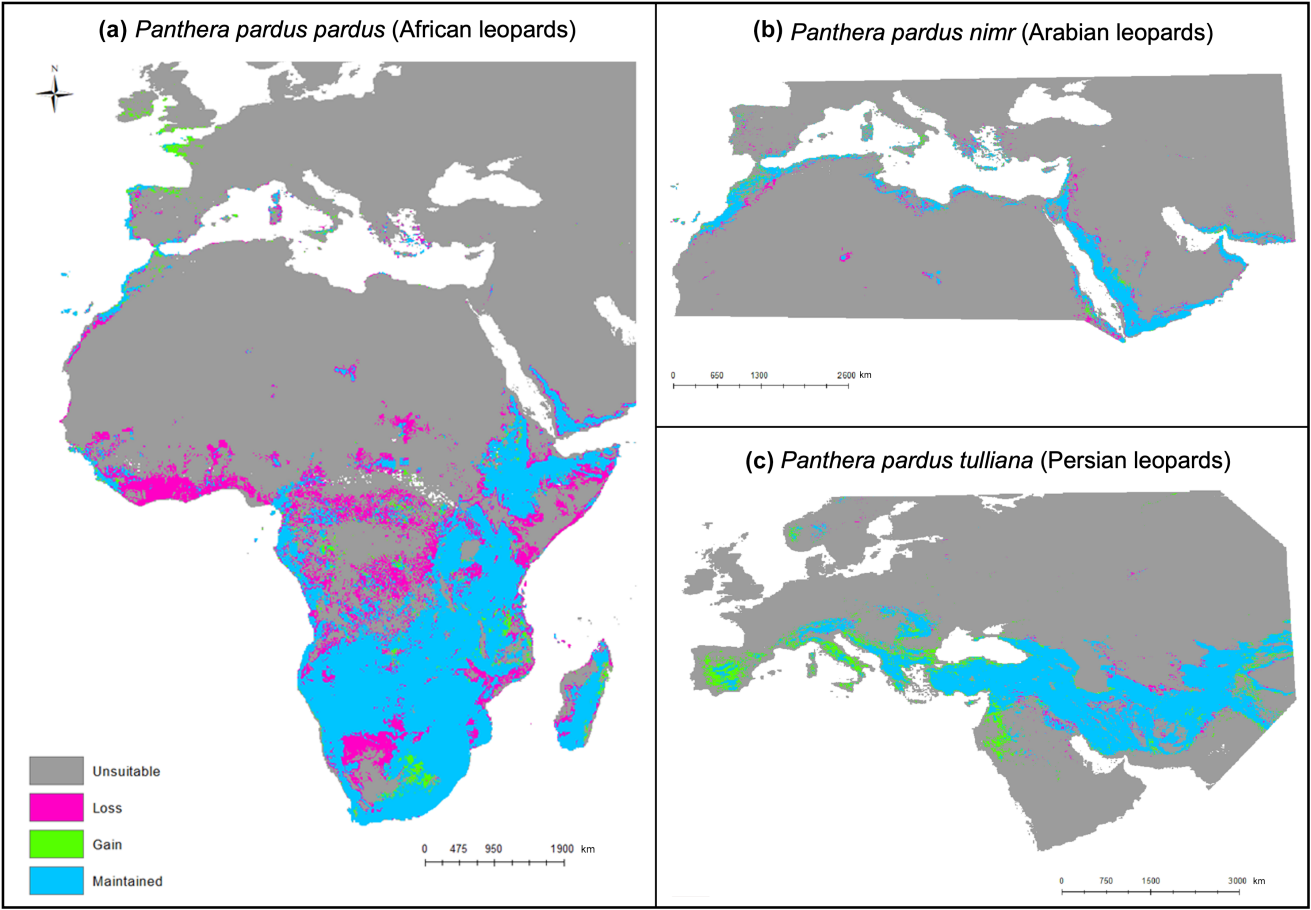
Changes in range suitability between present and future (2050) conditions based on species distribution modelling predictions for the (a) African, (b) Arabian and (c) Persian leopards (grey – unsuitable areas; pink – areas suitable under present conditions predicted to become unsuitable in the future; green – areas unsuitable under present conditions predicted to become suitable in the future; blue – areas predicted to remain suitable under present and future conditions).

The Arabian leopard model spanned the Mediterranean (including southern Europe and North Africa) to the Arabian Peninsula and Southern Iran. The Persian leopard model spanned Europe, including southern Scandinavia, and Asia, including Iran, up to the Indus River and Himalayas on the east (Figure 2).

### 2.4 Modelling procedures

SDMs were generated with Maxent (v3.4.4; Phillips et al., 2006). Following the recommendations in Merow et al. (2013), we used the R package ENMEval (Kass et al., 2021) to optimise model parameters, setting regularisation multiplier values between 1 and 5 and including the Linear, Quadratic, Product and Hinge features. The best fit model selected based on AIC scores (Warren & Seifert, 2011) included all features and regularisation 1 for the African leopard, LQH features and regularisation 1 for the Arabian leopard, and all features and regularisation 2 for the Persian leopard. A larger regularisation multiplier results in a more diffused, less localised prediction of distribution compared to a smaller regularisation parameter. Models were generated with 10,000 background data points and 10 cross‐validations using the Cloglog output. Model performance was determined based on area under the receiver operator curve (AUC) test scores. AUC test scores >0.8 are generally regarded as good model discrimination ability (Thuiller et al., 2005). We generated multivariate environmental similarity surfaces (MESS) plots to identify areas where future variable projections fall outside their contemporary ranges. We used the thresholding method that maximises training sensitivity plus specificity to generate binary maps (unsuitable vs. suitable areas). This method is suitable for presence only data and has a good discriminatory power (Liu et al., 2013).

### 2.5 Spatial analysis of model outputs

We used the raster calculator function in ArcGIS (v10.6; ESRI) to overlap the thresholded present and future modelling outputs and calculate the percent of range change for each leopard subspecies. We downloaded the Protected Areas map, WDPA_Feb2023, from the World Database on Protected Areas (UNEP‐WCMC, 2023), a comprehensive global database of marine and terrestrial PAs. We calculated the percent of predicted suitable areas falling inside PAs under current and future conditions for each subspecies and calculated percent differences in coverage between the two time periods. Model predictions were clipped to the known distribution for each subspecies based on the IUCN Red List of threatened species (downloaded from https://www.iucnredlist.org/species/15954/215195554).

## 3 RESULTS

All models performed well with high discrimination ability (average test AUC scores ranged between 0.859 ± 0.007 and 0.978 ± 0.007; Table 1). Models were not affected by variables outside their training range because areas impacted did not fall within predicted suitable ranges (Figure S1). Across all subspecies, temperature seasonality was a key variable affecting environmental suitability. Topographic ruggedness was important for Arabian and Persian leopards. Land use variable contribution differed between subspecies, but key variables included broadleaf forest cover, pasture (land covered with grass and other low plants suitable for grazing) and shrub (Table 1). Both African and Arabian leopards had similar responses to temperature seasonality, with higher occurrence probability at lower values and low occurrence probability at medium values, particularly for African leopards. In contrast, Persian leopards had high occurrence probability at medium values. Environmental suitability for African leopards also increased with maximum temperatures of the warmest month (BIO5; 29.5–30.7°C), while for Persian leopards it increased with mean temperatures of the driest quarter, peaking at 29.5°C. Topographic ruggedness was important for Persian and Arabian leopards, which favoured areas with medium‐high ruggedness. Shrub and pasture had a positive impact on environmental suitability for African and Arabian leopards, respectively (Figures S2–S4).

**TABLE 1:** Model evaluation and environmental layers included in the models for each subspecies and their percent contribution to the models (NA, not included in the model).

	*Panthera pardus pardus*	*Panthera pardus nimr*	*Panthera pardus tulliana*
Test AUC scores	0.859 ± 0.01	0.978 ± 0.01	0.930 ± 0.02
Temperature seasonality	41.1	25.8	14.2
Maximum temperature of warmest month	9.7	0.8	NA
Mean temperature of wettest quarter	2.3	1.4	4.7
Mean temperature of driest quarter	8.1	1.8	4.9
Annual precipitation	NA	2.9	NA
Precipitation of wettest month	1.5	NA	0.1
Precipitation seasonality	1.3	2.9	3.4
Precipitation of driest quarter	1.4	NA	0.8
Snow cover days	2.7	1.9	1.2
Ruggedness	3.4	41.9	61.5
Arable cover	NA	2	4
Broadleaf forest cover	0.3	6.5	NA
Coniferous forest cover	NA	1.7	NA
Grassland cover	0.1	1.9	NA
Pasture cover	1.9	6.4	1.4
Riparian cover	NA	NA	1.3
Shrub cover	21.5	NA	2.3
Sparse vegetation cover	3.7	2.2	NA
Urban cover	1	NA	NA
Water cover	NA	NA	0.2

### 3.1 Current suitable range predictions

Predicted suitable areas for the African leopard spanned across the majority of Sub‐Saharan Africa and Madagascar, as well as coastal parts of the Arabian Peninsula and the west coast of North Africa (Figure S5). For the Arabian leopard, suitable areas are predicted mostly in Western Saudi Arabia and Yemen, as well as along the Mediterranean coast of North Africa and Southern Iran (Figure S6). For the Persian leopard, predicted suitable areas spanned across Turkey, Iraq, and Iran, some parts of south‐eastern Europe and continued east to Pakistan and the Himalayas (Figure S7).

### 3.2 Future range change predictions

Under future climate and land‐use change scenarios, African and Arabian leopards are predicted to experience suitable range contractions, whereas Persian leopards are predicted to experience expansion of suitable range (Table 2). The African leopard is predicted to experience reduced environmental suitability and increased fragmentation in western and central Africa, particularly in the Democratic Republic of Congo and the Central African Republic. Southern suitable range contractions are predicted in Namibia and Botswana. In eastern Africa, suitable range contractions are predicted in Kenya. However, new suitable areas are predicted in Europe (Figure 2). Present and 2050 predictions had 71.40% overlap, but predicted suitable range decreased by 24.81% (Table 2). The Arabian leopard is predicted to experience slightly lower suitable range contraction (13.98%) primarily in Northern Africa, parts of Turkey, Greece, Syria, Jordan and Saudi Arabia. Suitable range gains are predicted in North Africa, particularly Morocco, some European countries and very limited in Saudi Arabia (Figure 2). In contrast, the Persian leopard is predicted to experience 11.81% suitable range gains and maintain a high suitable range overlap with present suitable range (94.59%). Suitable range gains are predicted in Saudi Arabia and southern Europe (Italy and Spain), while eastern parts of the range are predicted to experience slight loss of suitable range (Figure 2). When subspecies maps were clipped to their current known distribution, the overlap between current distribution and future predictions increased in the African leopard to 89.57% and decreased in Arabian and Persian leopards to 42.45% and 25.52%, respectively (Table 2).

**TABLE 2:** Predicted changes in suitable range for the three western leopard subspecies, including the percent of the study area predicted to be suitable under present and future (2050, rcp8.5) conditions, percent change in range suitability and percent range overlap between conditions.

	% present	% future (2050)	% change	% range change	% range overlap	% overlap future with known distribution
*P. p. pardus*	24.43	18.37	−6.06	−24.81	71.4	89.57
*P. p. nimr*	7.08	6.09	−0.99	−13.98	77.64	42.45
*P. p. tulliana*	17.27	19.31	2.04	11.81	94.59	25.52

### 3.3 Gap analysis: overlap with protected areas

**FIGURE 3:**
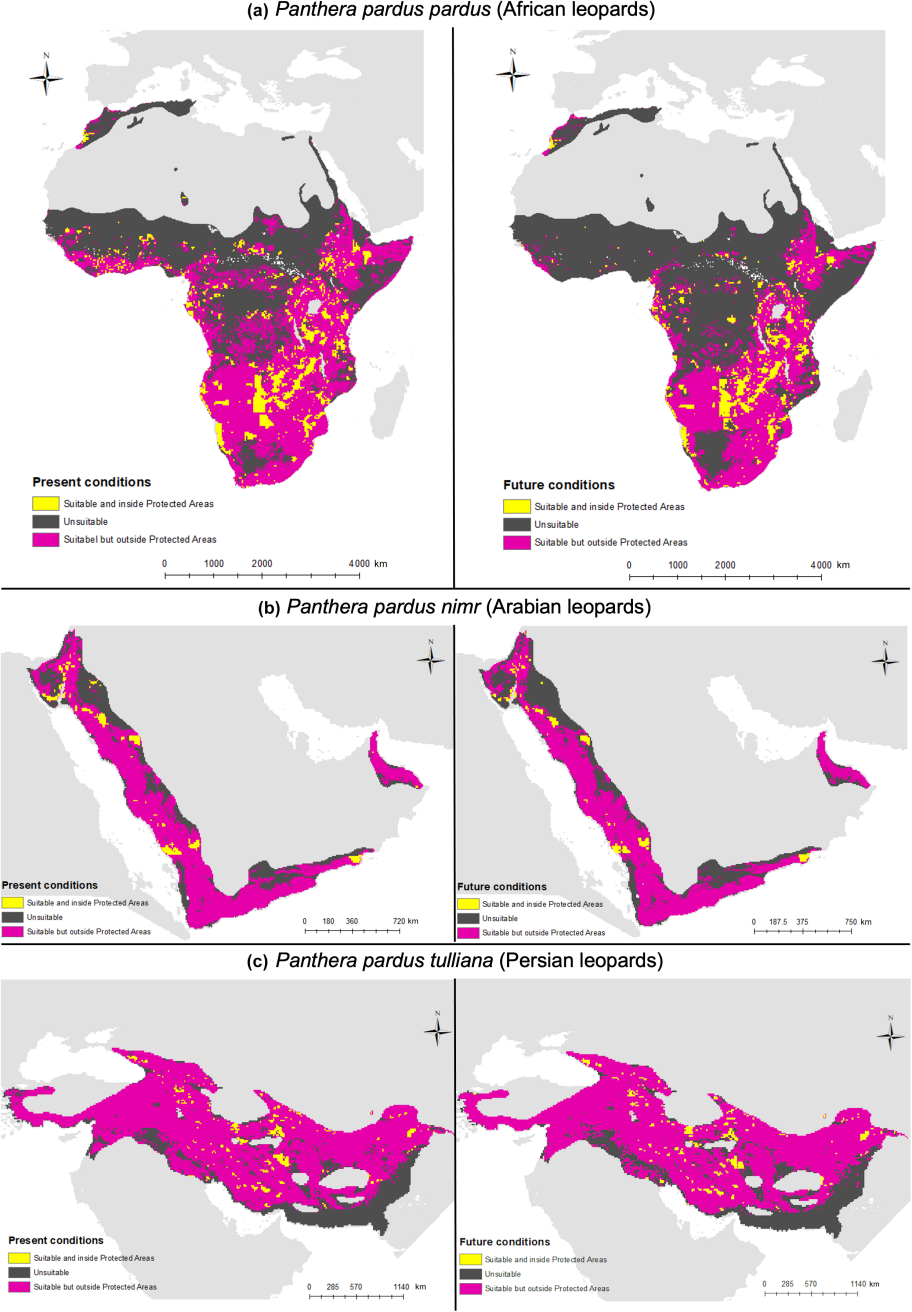
Overlap between predicted suitable range and protected areas under present (a, c, e) and future (2050; b, d, f) conditions for the African leopard (a, b), Arabian leopard (c, d), and Persian leopard (e, f). Model predictions were clipped to the current known range for each subspecies based on IUCN. Maps show predicted unsuitable areas in dark grey, suitable areas inside protected areas in yellow and suitable areas outside protected areas in pink.

Only a small percentage of the predicted current suitable leopard range falls inside PAs, 4.13% for the Persian leopard, 6.35% for Arabian and 16.58% for the African leopard (Figure 3). Overlap with PAs is predicted to decrease under future conditions for all subspecies, ranging from 20.85% decrease in the African leopard to 3.02% decrease in the Persian leopard (Table 3). Losses of predicted suitable range falling inside PAs are particularly evident in West Africa for the African leopard and northern parts of the distribution of the Arabian leopard (Figure 3).

**TABLE 3:** Percent of predicted leopard suitable areas under present and future (2050, rcp8.5) conditions falling within protected areas and percent change in overlap with protected areas between present and future conditions when clipped to the known distributions of the three subspecies based on the IUCN (Stein, 2020).

	% present overlap	% future overlap	% change
*P. p. pardus*	16.58	17.70	−20.85
*P. p. nimr*	6.35	5.61	−19.28
*P. p. tulliana*	4.13	4.03	−3.02

## 4 DISCUSSION

We identified climate and land cover variables that contribute to range suitability for three leopard subspecies (African, Persian and Arabian) and included these variables to predict future range suitability for the subspecies. We show that subspecies differ in their environmental associations and vulnerability to climate and land‐use change. Most concerningly, we found that the majority of areas predicted to be suitable for leopards were not protected despite the threatened conservation status of some leopard subspecies. Moreover, the proportion of suitable range inside PAs is predicted to decrease under climate and land‐use change, threatening global capacity to effectively conserve leopards across these regions.

### 4.1 Leopard subspecies differ in their environmental associations

Range suitability was governed by different variables for each subspecies, highlighting the importance of intraspecific modelling. Yet, some variables were important for all leopard subspecies. All subspecies had a low probability of occurrence at high levels of temperature seasonality, conditions common in continental temperate zones that have high fluctuations between summer and winter (Mosbrugger et al., 2005). This could explain why most leopards are found in tropical or subtropical zones. For example, African leopards have the highest probability of occurrence in tropical rainforest and savanna biomes, where temperatures remain relatively constant throughout the year (Alberts et al., 2009; Wesche et al., 2016). These habitats contain preferred land cover types, such as shrubs, forests and grassland, that provide optimal conditions for stalk hunting, concealment and refuge (Loveridge, Sousa, Seymour‐Smith, et al., 2022). However, suitable areas for the Persian leopard are predicted in more northerly latitudes, across southern Europe, linked to their association with medium levels of temperature seasonality. This demonstrates that certain subspecies may be suited to a range of environments that we may not currently associate with leopards, and highlights that areas suitable for one species will not necessarily be suitable for all.

Temperature was identified as an important variable in previous leopard and other large carnivore modelling studies, particularly maximum temperatures combined with rainfall (Farhadinia et al., 2015; Hosseini et al., 2019; Jones et al., 2016), likely linked to the effect of droughts on vegetation cover and prey abundance. Maximum temperature and rainfall affect environmental moisture content, which can lead to the evolution of distinct melanistic phenotypes in different leopard subspecies in areas with higher levels of moisture, likely due to an evolutionary advantage of this phenotype in dense vegetation cover (da Silva et al., 2017). In our study, rainfall variables had a weaker effect than temperature variables on model predictions, though their effect was stronger in the Arabian leopard, possibly linked to restricted water availability and the relationship between rainfall and increased vegetation cover in arid environments (Dunford et al., 2022; Olmos‐Trujillo et al., 2020).

For Persian and Arabian leopards, ruggedness most strongly influenced range suitability. Steep hillside habitats provide refuge from anthropogenic disturbance, reduced competition with humans and other competitors, and increased prey abundance (Khosravi et al., 2019; Sari, 2022). Ruggedness was identified as a key variable in previous modelling studies (Farhadinia et al., 2015; Kaboodvandpour et al., 2021; Loveridge, Sousa, Seymour‐Smith, et al., 2022). Given that leopards benefit from higher elevations in both rangelands and protected areas (Drouilly et al., 2018), conservation efforts should focus on rugged, mountainous areas. Dunford et al. (2022) found slope and ruggedness to be highly correlated and discussed the benefits of intermediate slopes and elevations. Intermediate slopes provide refuge from human disturbance and have lower associated energetic costs than steeper slopes (Dunford et al., 2022). Hence, it is not just the steepest terrains that should be considered viable habitat but also intermittent slopes and elevations that reduce energy expenditure.

The leopard subspecies differ in their land cover associations. African leopards are associated with areas with high shrub cover, likely due to better cover for hunting, increased prey availability and potential avoidance of other large carnivores, which may be more strongly associated with open landscapes (Loveridge, Sousa, Seymour‐Smith, et al., 2022). In contrast, Arabian leopards are associated with sparse vegetation and low forest cover, characteristics of arid and low human density environments. In arid landscapes people and livestock often gather in areas close to water sources reducing vegetation cover (Goirán et al., 2012) and pushing large carnivores away (Dunford et al., 2022). They are also associated with areas with high cover of grasslands suitable for pasture, which have higher forage quality and consequently increased prey, but also increased risk of human‐wildlife conflict due to predation on livestock (Bagheriyan et al., 2023). The Persian leopard model is less affected by land cover variables, and instead more influenced by ruggedness, indicating that this subspecies may be more flexible in its habitat use or may be more strongly affected by competition for prey or persecution in more accessible terrains. Despite these differences, the probability of occurrence of all subspecies declines with arable cover. Increasingly, land is being converted to agriculture, increasing fragmentation, reducing habitat quality and threatening wildlife populations, including prey (Foley et al., 2005). Although not a key contributor in this study, the threat will likely grow in the future due to growing economies and increasing food demands with expanding human populations (Tilman et al., 2001), leading to range losses among already threatened carnivores (Di Minin et al., 2016).

### 4.2 Leopard subspecies differ in their vulnerability to climate and land‐use changes

The African leopard is projected to experience the greatest suitable range reduction followed by the Arabian leopard, while the suitable range of the Persian leopard is predicted to increase under our model conditions. With Africa projected to experience above‐average climate change in the 21st century (Simmons et al., 2004), it is unsurprising that the African leopard is predicted to experience the greatest decline in range suitability. Temperature increases and longer, more intense dry seasons in tropical forests are causing increased droughts, wildfire risk and tree mortality (Wigneron et al., 2020). Reduced vegetation can negatively impact leopards, through increasing fragmentation and reducing herbivore prey availability.

The most prominent contributing factor to range suitability for the Persian leopard at 61.5% is ruggedness. Because this environmental factor will not change in the near future, the range of this species is likely to remain suitable. A previous study has also predicted range expansion for this species (Ebrahimi et al., 2021). Whilst the Persian leopard is predicted to gain suitable range under climate change, only a quarter of its current realised range overlaps with future suitable areas. Reintroductions and assisted migration through translocations and meta‐population management may be needed to help sustain the subspecies. However, high connectivity between currently occupied patches and historic regions appropriate for population recovery (Bleyhl et al., 2022) suggests leopards may not need human intervention to recolonise their historic ranges if connectivity can be maintained.

Leopards are not the only species to experience range contractions. Other large carnivores, such as lions (*Panthera leo*) and tigers (*Panthera tigris*), are predicted to experience similar range losses (Ebrahimi et al., 2021; Kc et al., 2020; Loveridge, Sousa, Seymour‐Smith, et al., 2022). Decreased connectivity is causing loss of genetic diversity and increasing isolation of populations (Loveridge, Sousa, Cushman, et al., 2022). Consequences are affecting whole communities, with reduced species ranges, changes in abundance, richness and diversity and reduced juvenile survival (Kupika et al., 2018).

Actual range losses could be less than projected as leopards have high adaptability and conservation efforts may help maintain leopard range (CMS, 2022). However, range loss could also be greater than projected because leopards are unlikely to occupy their full predicted future range due to missing or incompatible biotic interactions and dispersal limitations. Biotic factors, such as prey abundance, competition with other large carnivores and negative interactions with humans will also have a strong impact on distribution changes, but their importance may vary between the subspecies. The African leopard could experience increased competition with other, more dominant carnivores, such as lions and hyenas (*Hyaenidae* spp.), whereas Arabian and Persian leopards are considered apex predators across their range (Hebblewhite et al., 2011; Zafar‐ul Islam et al., 2021). Previous large carnivore models have incorporated the distribution of relevant prey species (Ebrahimi et al., 2017). However, this may be challenging for leopards due to their broad, generalist diet. Mechanistic models (Jarvie & Svenning, 2018) can provide more accurate range change projections because they take species physiology, demography and dispersal behaviour into consideration, but the detailed life history and functional trait data they require are missing for most species (Urban et al., 2016). Model predictive ability may be further limited by location data bias (Beck et al., 2014) and limited data availability from under‐sampled areas, such as west and central African rainforest regions.

Variable responses between subspecies are not uncommon; however, this study highlights how drastic these differences can be, thus reiterating the importance of considering intraspecific variation when modelling vulnerability to climate and land‐use changes. Similar differences in model projections are found between subspecies of large mammals, birds and locusts (Meynard et al., 2017; Vásquez‐Aguilar et al., 2021; Wan et al., 2019). Historically all subspecies of leopards have experienced different range changes. The Arabian leopard is currently occupying only 2% of its historic range, having experienced the greatest range loss of all subspecies, yet the African leopard has been extirpated from the greatest number of locations (Jacobson et al., 2016). Identified differences in vulnerability to future climate and land‐use changes support the need to manage and assess these subspecies separately. Furthermore, populations of subspecies distributed over large geographic areas that experience different climatic and land‐use conditions would also benefit from being assessed separately. For example, African leopard populations in southern, central, eastern and west Africa.

### 4.3 Conservation gaps

This study highlights the small proportion of the suitable realised range of the Arabian and Persian leopards within PAs (ca. 4%–6%) partly due to limited PA coverage in the Middle East (Omari, 2011). Higher overlap (17%) identified in previous assessments considering the species as a whole (Jacobson et al., 2016), further supports our call for intraspecific assessment and conservation management. Limited PA coverage is concerning given that PAs are considered the last strongholds for many threatened mammals (Pacifici et al., 2020). We show that the proportion of suitable range falling inside PAs will likely decrease for the three subspecies over the coming decades, highlighting the importance of conserving leopards in the wider landscape outside PAs (Di Minin et al., 2016).

Leopards require larger habitat patches to cover their large home ranges and better‐connected patches to ensure genetic exchange and sustain larger populations that are less sensitive to extirpations (Bleyhl et al., 2021). For African leopards, habitat restoration and improvement outside PAs in eastern Africa, where models predict maintained range suitability, and north‐western Africa, where potential range gains are predicted, is needed to increase landscape connectivity and facilitate gene flow between isolated populations. The Arabian leopard is divided into multiple small subpopulations, further increasing the risk of inbreeding and extirpation (Jacobson et al., 2016). The subspecies is currently at high risk of becoming Critically Endangered with population estimates of 100–250 adults remaining in the wild (Al‐Johany, 2007) and no individuals left in Saudi Arabia (Dunford et al., 2024).

Large carnivore persecution is a major cause of population decline (Bleyhl et al., 2021). Survival rates for leopards are significantly higher in PAs (Swanepoel et al., 2015), with most deaths outside of PAs attributed to deliberate killings by humans for body parts, conflict with livestock or due to them being perceived to be dangerous (Bleyhl et al., 2021; Swanepoel et al., 2015). Persecution can be reduced through implementing conflict mitigation measures and promoting alternative husbandry methods to prevent livestock depredation (Balme et al., 2009). Depletion of leopard prey, such as gazelles and ibex, through hunting by humans outside PAs (Stein, 2020) poses an indirect threat (Wolf & Ripple, 2016) forcing leopards to increase their home ranges and causing population declines (Hayward et al., 2007). Leopards might respond by preying on livestock, leading to them being perceived as pests, hunted or poisoned (Al‐Johany, 2007; Drouilly et al., 2023; Parchizadeh & Belant, 2021; Soofi et al., 2022). A strong reduction in persecution alongside prey restoration can restore leopard populations by giving them the chance to recolonise patches. However, in the absence of conservation strategies, local populations became extinct despite reduced persecution (Bleyhl et al., 2021). It is important that conservation efforts span across both private and public land and integrate conservation actions with other land uses, such as farming, through working together with different stakeholders (Norton, 2000).

### 4.4 Conservation opportunities

Our models predict that large areas of Europe could become climatically suitable for leopards by 2050, particularly for the Persian leopard. During the Pleistocene, several large carnivores were found in Europe, including wolves, *Canis lupus*, bears, *Ursus spelaeus* and *Ursus arctos*, leopards, *Panthera pardus*, lions, *Panthera fossilis* and *Panthera spelaea*, and hyena, *Crocuta crocuta spelaea* (Masseti & Mazza, 2013; Paijmans et al., 2018). The little‐known European Ice‐Age leopard, *P. p. spelaea*, occurred across Europe until 17,000 years ago, southern Europe until ~11,000 ya and in the Balkans until 9000 ya (Sommer & Benecke, 2006). The occurrence of these extinctions millennia after the Last Glacial Maximum, despite abundant habitat and prey availability, points to humans as a likely cause of these extinctions (Sommer & Benecke, 2006).

This likely increase in climatic suitability throughout Europe, in combination with projected suitable range losses in other parts of the current leopard range, especially for the Persian leopard, may open up opportunities for expansion outside of their contemporary range. Although lack of connectivity may limit colonisation of newly suitable areas, there are initiatives experimenting with large‐scale translocations to restore ecological roles, which were previously lost due to local extinctions (e.g. Briers‐Louw et al., 2019). Rewilding has recently emerged as a paradigm shift in the way conservation and nature are viewed, emphasising complete, functional and robust ecosystems, and restoring natural cycles of water, nutrients and energy (Pereira & Navarro, 2015). Pleistocene rewilding aims to address the disproportionate loss of ecologically dominant and influential keystone megafauna following human expansion across the globe through reintroductions or translocations of species that perform similar ecological functions to previously present large fauna (Donlan et al., 2006; Zimov, 2005). European rewilding currently focuses on restoring large herbivore populations, but establishing prey bases also provides an opportunity for the return of carnivores, popularised by the success of wolf reintroduction to America's Yellowstone National Park (Ripple & Beschta, 2012). For example, the Eurasian lynx has been reintroduced across Europe (Linnell et al., 2009) to Poland (Skorupski et al., 2022) and Slovenia, from where they were extirpated in the early 20th century (Kos et al., 2012). There is also discussion about reintroducing lynx to its former range in Scotland (Bavin et al., 2023; Ovenden et al., 2019). However, carnivore translocations can face challenges, primarily due to reported anthropogenically caused mortality (Stepkovitch et al., 2022), stressing the importance of considering implications for farmers, hunters and the forestry sector (Drouilly & O'Riain, 2021). The perception of lynx, wolves and other carnivores varies between stakeholders (Van Heel et al., 2017) and can change rapidly (Niemiec et al., 2022), illustrating the complexities of carnivore return and reintroductions. However, with proper stakeholder involvement and increasing rural depopulation, Europe could, at least theoretically, provide possible sites for range expansion of the Persian leopard in areas identified by our models as suitable under future conditions.

Among large felids, leopards may be the best candidate for expansion into Europe, despite lions persisting in Europe for longer (Masseti & Mazza, 2013). Leopards are solitary, with a wide niche breadth and are highly adaptable, even in human‐dominated landscapes (Athreya et al., 2016; Braczkowski et al., 2018; Stein et al., 2011). Whilst the presence of other large carnivores could potentially negatively affect leopard densities, the major challenges facing European expansion may include human acceptance, connectivity and prey availability (Ebrahimi et al., 2017). As seen in the Eurasian lynx, illegal killings, road collisions and low genetic diversity due to a lack of connectivity and small founder populations may also prove challenging (Iannella et al., 2024; Sindičić et al., 2013; Skorupski et al., 2022). The Persian leopard is in particular need of finding new suitable areas to ensure its long‐term survival. Only a quarter of its current range overlaps with predicted future suitable areas and only 4% of that range falls within PAs. Under such projected range changes, one approach would be to explore opportunities for assisted translocations outside the current range to safeguard leopard populations for the future. However, people are more likely to accept leopards in Europe if they recolonised naturally without human intervention (Lüchtrath & Schraml, 2015), though this may vary between social groups and sectors (Whiley & Tzanopoulos, 2024). Hence, efforts to increase prey availability and connectivity to encourage leopard recolonisation of habitats will likely be more successful than human‐led translocations.

### 4.5 Limitations

Whilst our models highlight areas that would potentially benefit from conservation efforts it is important to acknowledge that it is unlikely leopards will range across the full predicted range. This is due to future changes in biotic interactions and additional biotic and abiotic factors not considered in the models. SDMs assume that any predicted suitable habitat can be occupied, which is unrealistic. Model predictions can be improved by integrating dispersal and dispersal pathways (Araújo & Guisan, 2006); however, these are difficult to predict (Elith & Leathwick, 2009).

In addition, our models do not consider one of the biggest challenges facing leopards, the human dimension (Drouilly & O'Riain, 2021). The growing human population will have substantial impacts on biodiversity due to expanding human settlements and agriculture (Pacifici et al., 2015). Moreover, human perception and acceptance will influence the success of large carnivore recovery and range expansion (Drouilly & O'Riain, 2021). As with all carnivores, human conflict should be a main consideration when assessing conservation actions (Bodasing, 2022; Fernández‐Sepúlveda & Martín, 2022; Johnson et al., 2023; Ripple et al., 2014), and in the leopard's case, prey availability will influence rate of attacks on livestock, and consequently leopard‐human conflict (Jacobson et al., 2016).

## 5 CONCLUSIONS

Our study highlights the importance of considering intraspecific variation when assessing the predicted impacts of climate and land‐use change. Through modelling range suitability for three leopard subspecies, we show that subspecies differ not only in their environmental associations but also in their relative vulnerability to future changes. The inclusion of biotic interactions, dispersal behaviour, evolutionary adaptations and the human dimension may further improve model performance.

Leopards are classed as Vulnerable (Stein, 2020); yet, our study shows that only a small percentage of their suitable ranges fall within PAs, especially for Arabian and Persian leopards. The planned expansion of PAs can help reduce conflict with humans, as long as they involve local communities in their design and management (Farashi & Shariati, 2018). The possibility of European rewilding offers a further avenue for leopard conservation in a changing world. However, conservation efforts should focus on working with local communities across leopard ranges to convey the importance of apex predators and develop mitigation strategies based on local context. Examples include compensation schemes for those affected by leopards to reduce human‐wildlife conflict (Zeng et al., 2022), though the success of these schemes varies. Most importantly mitigation strategies should be specific to local needs and considered on a case‐by‐case basis.

Implementing conservation strategies for large carnivores is challenging and costly as protection must cover large, international and highly variable landscapes, requiring conservation interventions to be well‐planned and targeted (Bleyhl et al., 2021). Our models can help inform where to optimally allocate limited available conservation resources for maximum impact and identify populations at greatest risk. Based on our results, conservation efforts should focus on increasing native vegetation cover in Africa and protecting mountainous habitats for Persian and Arabian leopards. Habitat restoration and improvement outside PAs to increase landscape connectivity is particularly important across the ranges of the Arabian and Persian leopards, where PA coverage is low. Additionally, it is important to consider the human dimension and how human perceptions and conflict will influence the success of conservation actions.

## AUTHOR CONTRIBUTIONS

**Charlotte Mitchell:** Conceptualization (equal); formal analysis (lead); writing – original draft (lead). **Jamie Bolam:** Conceptualization (equal); data curation (lead); formal analysis (supporting); methodology (equal); writing – original draft (supporting); writing – review and editing (equal). **Laura D. Bertola:** Data curation (supporting); resources (equal); supervision (supporting); writing – review and editing (equal). **Vincent N. Naude:** Resources (equal); writing – review and editing (equal). **Lucas Gonçalves da Silva:** Resources (equal); writing – review and editing (equal). **Orly Razgour:** Conceptualization (equal); formal analysis (supporting); methodology (equal); project administration (lead); supervision (lead); writing – original draft (supporting); writing – review and editing (equal).

## CONFLICT OF INTEREST STATEMENT

Authors declare no conflict of interest.

## Supporting information


Data S1.

Data S2.

## Data Availability

Location records used in the modelling are available as Supporting Data file Data S1. Data sources are listed in the Supplementary Materials.
