# Supplementary material for: Leopard subspecies conservation under climate and land‐use change
Source: Ecol Evol. 2024 May 21;14(5):e11391. doi: 10.1002/ece3.11391 (PMC11109047; doi:10.1002/ece3.11391)
Supplement: Supplementary file 2 — Data S2. [file ECE3-14-e11391-s002.pdf]

Supplementary Information for

Leopard subspecies conservation under climate and land-use change

Supplementary Methods

List of publications used to extract location records data for the leopard subspecies:

Arabian leopard

Al-Johany, A.M.H., 2007. Distribution and conservation of the Arabian Leopard Panthera pardus nimr in Saudi Arabia. *Journal of Arid Environments*, *68*(1), pp.20-30.

Biquand, S., 1990. Short review of the status of the Arabian leopard, Panthera pardus nimr, in the Arabian Peninsula. *Unpublished Report of NWRC. Taif, Saudi Arabia*.

Biquand, S. and Boug, A., 1992. An update of leopard status in Al Fiqrah and recommendations for immediate action. *Report, NWRC, Taif*.

Budd K (1999) Short survey on the Arabian Leopard in the Kingdom of Saudi Arabia. Report.

da Silva LG, Kawanishi K, Henschel P, Kittle A, Sanei A, Reebin A, et al. (2017) Mapping black panthers: Macroecological modeling of melanism in leopards (Panthera pardus). *PLoS ONE* 12(4): e0170378.

Gasperetti J and Jackson P (1990) Preliminary Report of the Status of the Arabian Leopard. Unpublished report.15 pp. NWRC, Saudi Arabia.

Jacobson, A.P., Gerngross, P., Lemeris Jr, J.R., Schoonover, R.F., Anco, C., Breitenmoser-Würsten, C., Durant, S.M., Farhadinia, M.S., Henschel, P., Kamler, J.F. and Laguardia, A., 2016. Leopard (Panthera pardus) status, distribution, and the research efforts across its range. *PeerJ*, *4*, p.e1974

Judas, J., Paillat, P., Khoja, A. and Boug, A., 2006. Status of the Arabian leopard in Saudi Arabia. *Cat News*, *1*(1), pp.11-19.

Lagrot, I. and Lagrot, J.F., 1999. Leopard in the Arabian peninsula. *Cat News*, *30*, pp.21-22.

McGregor T, Spalton A, Al Hikmani H and Hammer M (2007) Status of the Arabian leopard (Panthera pardus nimr) in the mountains of the Musandam Peninsula, Sultanate of Oman. Report to Biosphere Expeditions.

Nader, I.A., 1989. Rare and endangered mammals of Saudi Arabia. *Wildlife Conservation and Development in Saudi Arabia*, *3*, pp.220-233.

Paillat P and Khoja A (1998) Incidental Studies – Arabian Leopard Panthera pardus nimr. Unpublished annual report, NWRC, Taif, Saudi Arabia.

Perez, I., Geffen, E. and Mokady, O., 2006. Critically endangered Arabian leopards Panthera pardus nimr in Israel: estimating population parameters using molecular scatology. *Oryx*, *40*(3), pp.295-301.

Smith TR (1993) Memorandum: Trip from 3–6 May 1993 to Investigate Report of Leopards. Unpublished report, NWRC, Taif, Saudi Arabia.

Spalton, J.A., Al Hikmani, H.M., Willis, D. and Said, A.S.B., 2006. Critically Endangered Arabian leopards Panthera pardus nimr persist in the Jabal Samhan Nature Reserve, Oman. *Oryx*, *40*(3), pp.287-294.

Zafar-ul Islam, M., Boug, A., Judas, J. and As-Shehri, A., 2018. Conservation challenges for the Arabian Leopard (Panthera pardus nimr) in the Western Highlands of Arabia. *Biodiversity*, *19*(3-4), pp.188-197.

Zafar-ul Islam, M., Gavashelishvili, A., Kokiashvili, L., al Boug, A. and Shehri, A.A., 2021. Modeling the distribution and movement intensity of the Arabian Leopard Panthera pardus nimr (Mammalia: Felidae). *Zoology in the Middle East*, *67*(2), pp.106-118.

Persian Leopard

Askerov, E., Talibov, T., Manvelyan, K., Zazanashvili, N., Malkhasyan, A., Fatullayev, P. and Heidelberg, A., 2015. South-Eastern Lesser Caucasus: the most important landscape for conserving the Leopard (Panthera pardus) in the Caucasus region (Mammalia: Felidae). *Zoology in the Middle East*, *61*(2), pp.95-101.

Avgan, B., Huseynali, T.T., Ismayilov, A., Fatullayev, P., Askerov, E. and Breitenmoser, U., 2012. First hard evidence of leopard in Nakhchivan. *Cat News*, *57*, p.33.

Avgan, B., Raza, H., Barzani, M. and Breitenmoser, U., 2016. Do recent leopard Panthera pardus records from northern Iraq and south-eastern Turkey reveal an unknown population nucleus in the region?. *Zoology in the Middle East*, *62*(2), pp.95-104.

Breitenmoser, U., Askerov, E., Soofi, M., Breitenmoser-Würsten, C., Heidelberg, A., Manvelyan, K. and Zazanashvili, N., 2017. Transboundary leopard conservation in the Lesser Caucasus and the Alborz Range. *Cat News*, *65*, pp.24-25.

da Silva, L.G., Kawanishi, K., Henschel, P., Kittle, A., Sanei, A., Reebin, A., Miquelle, D., Stein, A.B., Watson, A., Kekule, L.B. and Machado, R.B., 2017. Mapping black panthers: Macroecological modeling of melanism in leopards (Panthera pardus). *PLoS One*, *12*(4), p.e0170378.

gulfnews.com. (2021). *Rare Persian leopard pair sighted in Pakistan*. [online] Available at: https://gulfnews.com/world/asia/pakistan/rare-persian-leopard-pair-sighted-in-pakistan-1.1621595785073 [Accessed 22 Sep. 2023]

Jacobson, A.P., Gerngross, P., Lemeris Jr, J.R., Schoonover, R.F., Anco, C., Breitenmoser-Würsten, C., Durant, S.M., Farhadinia, M.S., Henschel, P., Kamler, J.F. and Laguardia, A., 2016. Leopard (Panthera pardus) status, distribution, and the research efforts across its range. *PeerJ*, *4*, p.e1974

Karataş, A., Bulut, Ş. and Akbaba, B., 2021. Camera trap records confirm the survival of the Leopard (Panthera pardus L., 1758) in eastern Turkey (Mammalia: Felidae). *Zoology in the Middle East*, *67*(3), pp.198-205.

Khorozyan, I.G. and Abramov, A.V., 2007. The Leopard, Panthera pardus,(Carnivora: Felidae) and its resilience to human pressure in the Caucasus. *Zoology in the Middle East*, *41*(1), pp.11-24.

Khorozyan, I.G., Cazon, A., Malkhasyan, A.G. and Abramov, A.V., 2007. Using thin-layer chromatography of fecal bile acids to study the leopard (Panthera pardus ciscaucasica) population. *Biology Bulletin*, *34*, pp.361-366.

Maharramova E., Moqanaki E. M., Askerov E., Faezi S., Alinezhad H., Mousavi M., Kuemmerle T., Heidelberg A. & Zazanashvili N. 2018. Transboundary leopard movement between Azerbaijan and Iran in the Southern Caucasus. *Cat News,* 67, 8-10.

Pestov, M.V., Nurmukhambetov, Z.E., Munkhashov, A.T., Terentyev, V.A. and Rosen, T. 2019. First camera trap record of Persian leopard in Ustyurt State Nature Reserve, Kazakhstan. *Cat News*, *69*, pp.14-16

Spassov, N., Askerov, E., Akosta-Pankov, I. and Ignatov, A., 2019. New data on the occurrence of Panthera pardus in the Talysh Mountains, Azerbaijan (Carnivora: Felidae). *Lynx, ns (Praha)*, 50, pp.113-118.

Toyran, K., 2018. Noteworthy record of Panthera pardus in Turkey (Carnivora: felidae). *Fresenius Environmental Bulletin*, *27*(11), pp.7348-7353.

Yarovenko, Y., 2010. Status and distribution of leopards (Panthera pardus) in the mountains of Dagestan, Russia

Yarovenko, Y. and Zazanashvili, N., 2016. Recent hard evidence for the occurrence of the Leopard, Panthera pardus (Mammalia: Felidae), in the Eastern Greater Caucasus. *Zoology in the Middle East*, 62(1), pp.88-90.

African leopard

da Silva, L.G., Kawanishi, K., Henschel, P., Kittle, A., Sanei, A., Reebin, A., Miquelle, D., Stein, A.B., Watson, A., Kekule, L.B. and Machado, R.B., 2017. Mapping black panthers: Macroecological modeling of melanism in leopards (Panthera pardus). *PLoS One*, 12(4), p.e0170378.

Eniang, E.A., Akani, G.C., Amadi, N., Dendi, D., Amori, G. and Luiselli, L., 2016. Recent distribution data and conservation status of the leopard (Panthera pardus) in the Niger Delta (Nigeria). *Tropical Zoology*, 29(4), pp.173-183.

Jacobson, A.P., Gerngross, P., Lemeris Jr, J.R., Schoonover, R.F., Anco, C., Breitenmoser-Würsten, C., Durant, S.M., Farhadinia, M.S., Henschel, P., Kamler, J.F. and Laguardia, A., 2016. Leopard (Panthera pardus) status, distribution, and the research efforts across its range. *PeerJ*, 4, p.e1974.

Naude, V.N., 2020. Scale and impact of the illegal leopard skin trade for traditional use in southern Africa. PhD thesis, submitted to the University of Cape town, South Africa. http://hdl.handle.net/11427/32936.

Soultan, A., Attum, O., Hamada, A., Hatab, E.B., Ahmed, S.E., Eisa, A., Al Sharif, I., Nagy, A. and Shohdi, W., 2017. Recent observation for leopard Panthera pardus in Egypt. *Mammalia*, 81(1), pp.115-117.

Westerberg, M., Craig, E. and Meheretu, Y., 2017. First record of African leopard (Panthera pardus pardus L.) in semi-arid area of Yechilay, northern Ethiopia. African *Journal of Ecology*, 56(2), pp.375-377.

Supplementary Tables

Table S1 – Description of environmental layers used in the models and their sources.

	Variable	Abbreviation	Description	Source
**Climate**	Temperature seasonality	BIO4	Standard deviation of the monthly mean temperatures (°C)	Chelsa-climate (https://chelsa-climate.org/)
	Maximum temperature of warmest month	BIO5	The highest temperature of any monthly daily mean maximum temperature (°C)	
	Mean temperature of wettest quarter	BIO8	The wettest quarter of the year is determined (to the nearest month) (°C)	
	Mean temperature of driest quarter	BIO9	The driest quarter of the year is determined (to the nearest month) (°C)	
	Annual precipitation	BIO12	Accumulated precipitation amount over 1 year	
	Precipitation of wettest month	BIO13	The precipitation of the wettest month	

	Precipitation seasonality	BIO15	The Coefficient of Variation is the standard deviation of the monthly precipitation estimates expressed as a percentage of the mean of those estimates.	
	Precipitation of driest quarter	BIO17	Rainfall during the driest quarter of the year.	
	Snow cover days	SCD	Number of days with snow cover.	
**Land cover**	Arable cover	Arable	Land worked (ploughed or tilled) regularly	Globio4 land cover map 2015 (Schipper, 2020).
	Broadleaf forest cover	Forest_bl	Composed of a mix of deciduous trees species and evergreen	
	Coniferous forest cover	Forest_con	Composed of conifers, trees that grow needles and cones	
	Grassland cover	Grassland	Land covered with grass.	

	Pasture cover	Pasture	Land covered with grass and other low plants suitable for grazing animals	
	Riparian cover	Riparian	Wetlands adjacent to rivers and streams	
	Shrub cover	Shrub	A woody small branching from or near the ground	
	Urban cover	Urban	Built up areas	
	Water cover	Water	Water resources	
**Topography**	Ruggedness	Ruggedness	Uneven land, representing mountainous areas.	Generated from the 30 arc sec elevation map from Worldclim (www.worldclim.org/)

Supplementary Figures

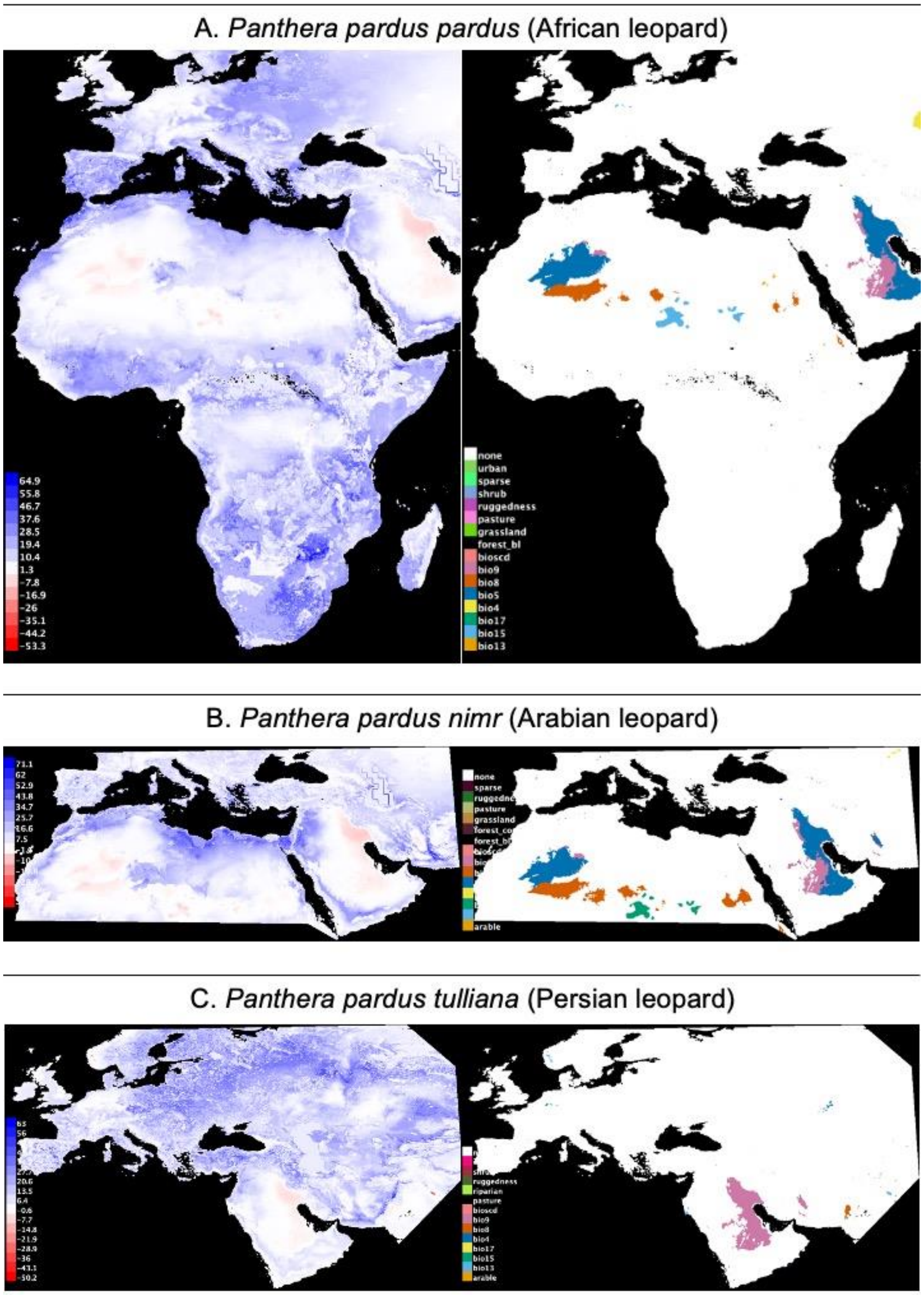

Figure S1 - Multivariate Environmental Similarity Surfaces (MESS) plots output from Maxent for A) *Panthera pardus pardus* (African leopards), B) *Panthera pardus nimr* (Arabian leopards), and C) *Panthera pardus tulliana* (Persian leopards). Plots on the left show areas where projected future variables are outside their present range (blue - within present range, red - outside). Plots on the right show the variables that are outside their present range.

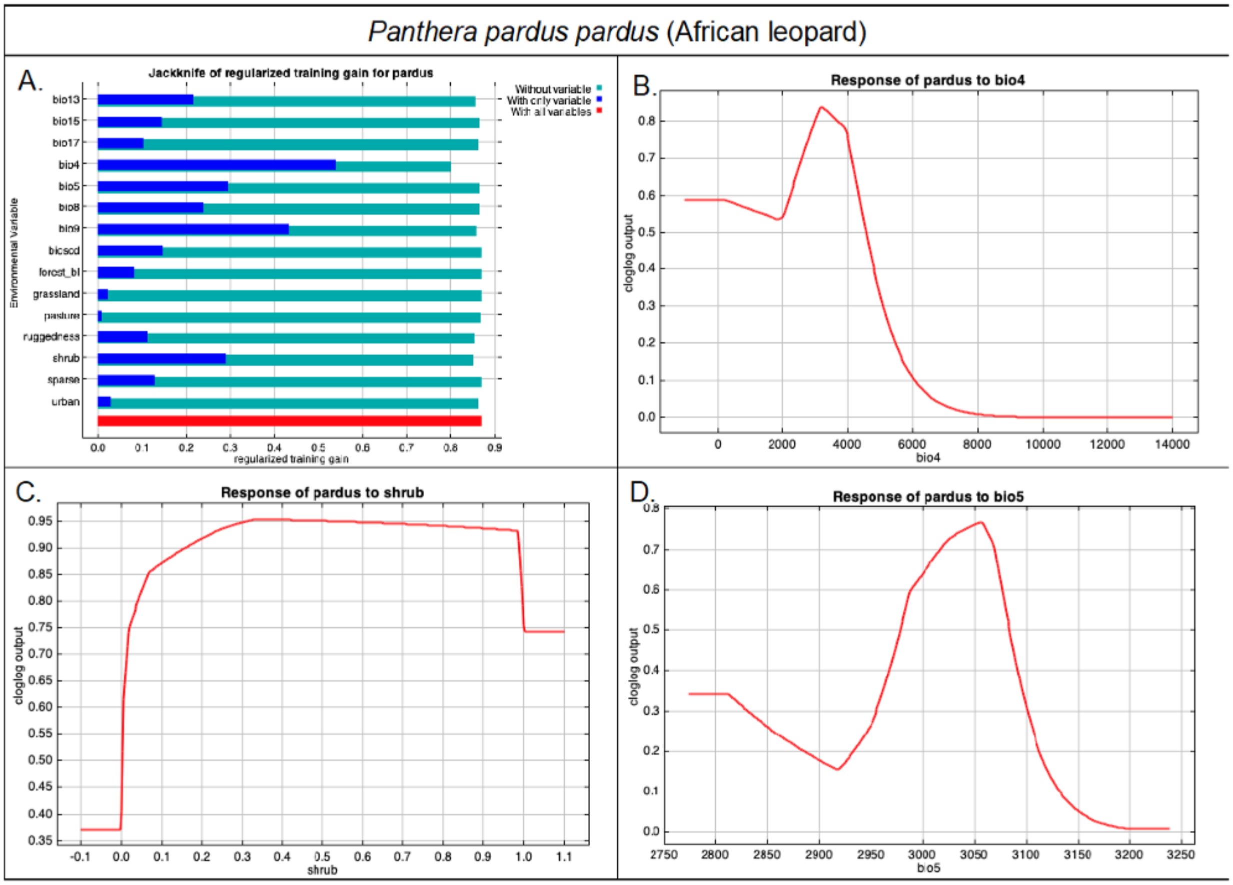

Figure S2 – Maxent modelling outputs for *Panthera pardus pardus* (African leopards). Jackknife plots of variable contribution to the model gain (A) and individual response curves of the most important environmental factors for each subspecies. B. Temperature seasonality, C. Percentage of shrub cover, D. Maximum temperature of warmest month.

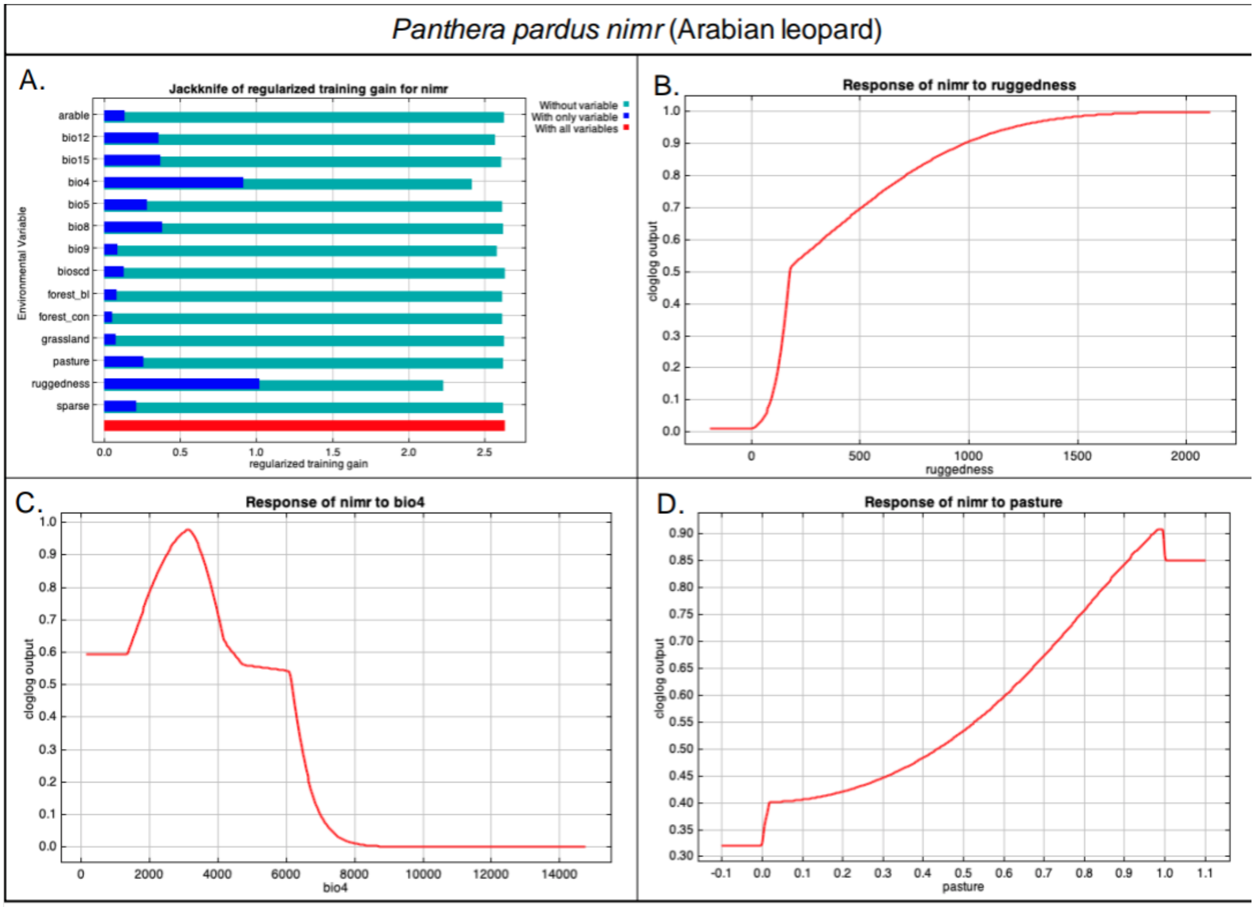

Figure S3 – Maxent modelling outputs for *Panthera pardus nimr* (Arabian leopard). Jackknife plots of variable contribution to the model gain (A) and individual response curves of the most important environmental factors for each subspecies. B. Ruggedness, C. Temperature seasonality, D. Percentage of pasture cover.

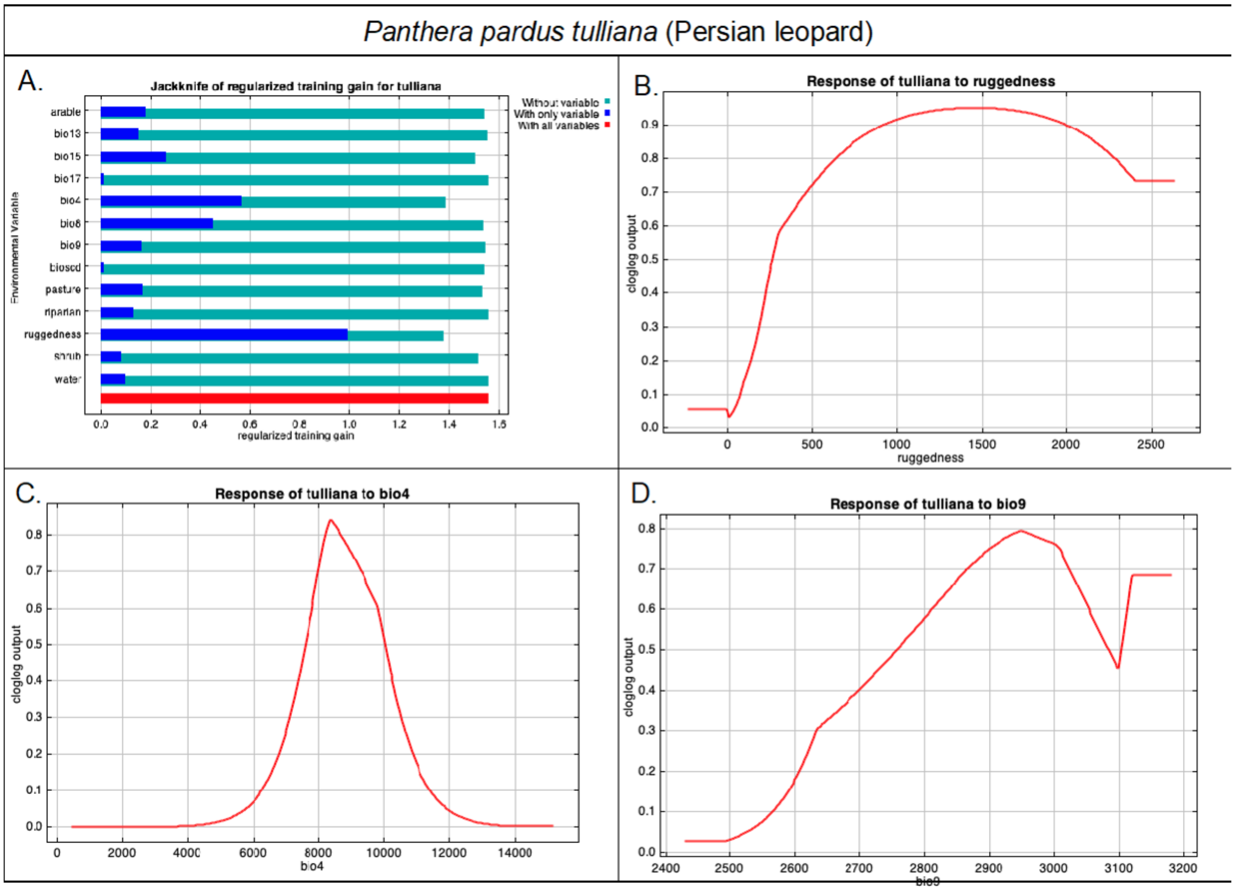

Figure S4 – Maxent modelling outputs for *Panthera pardus tulliana* (Persian leopards). Jackknife plots of variable contribution to the model gain (A) and individual response curves of the most important environmental factors for each subspecies. B. Ruggedness, C. Temperature seasonality, D. Mean temperature of driest quarter.

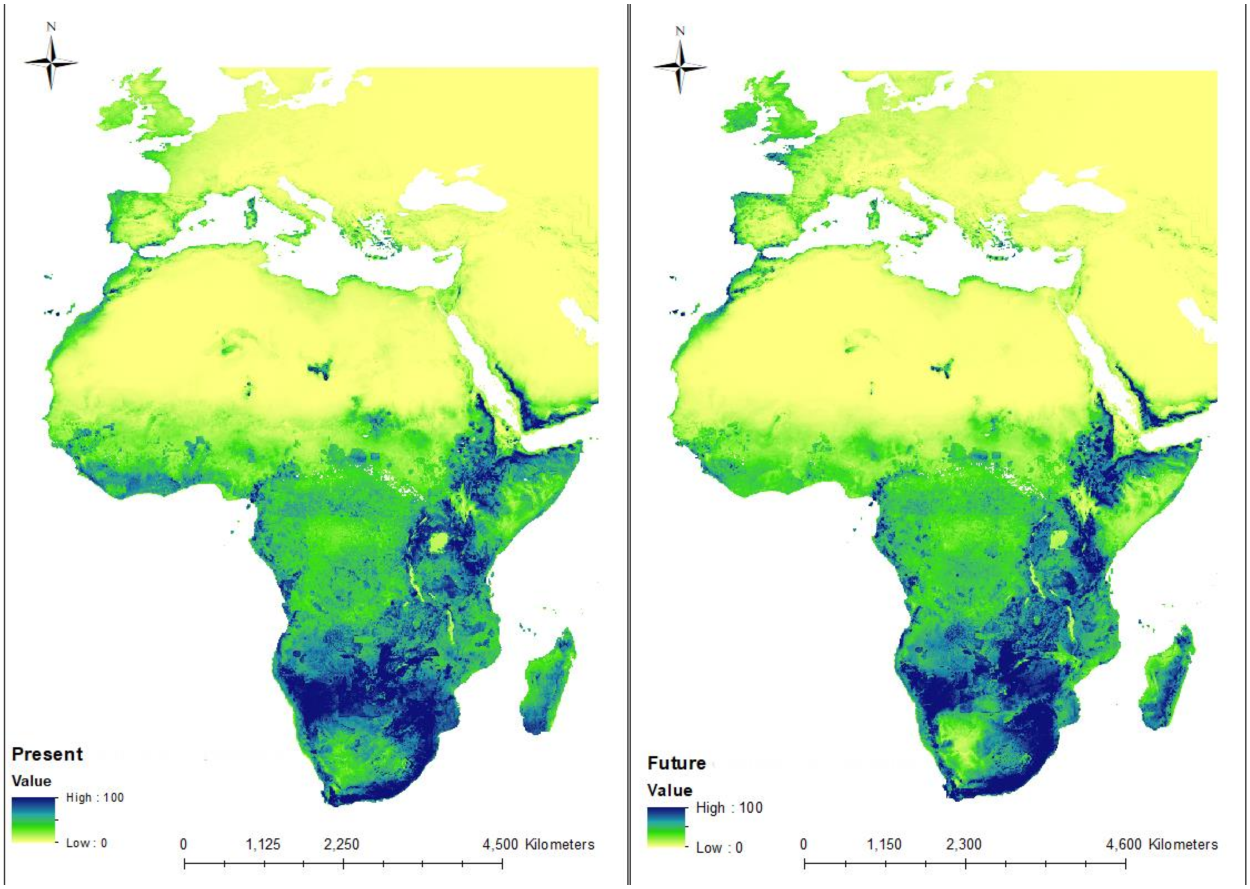

Figure S5 - Modelling output continuous maps showing predicted environmental suitability for *Panthera pardus pardus* (African leopards) under present (left) and future (2050, ssp585; right) conditions.

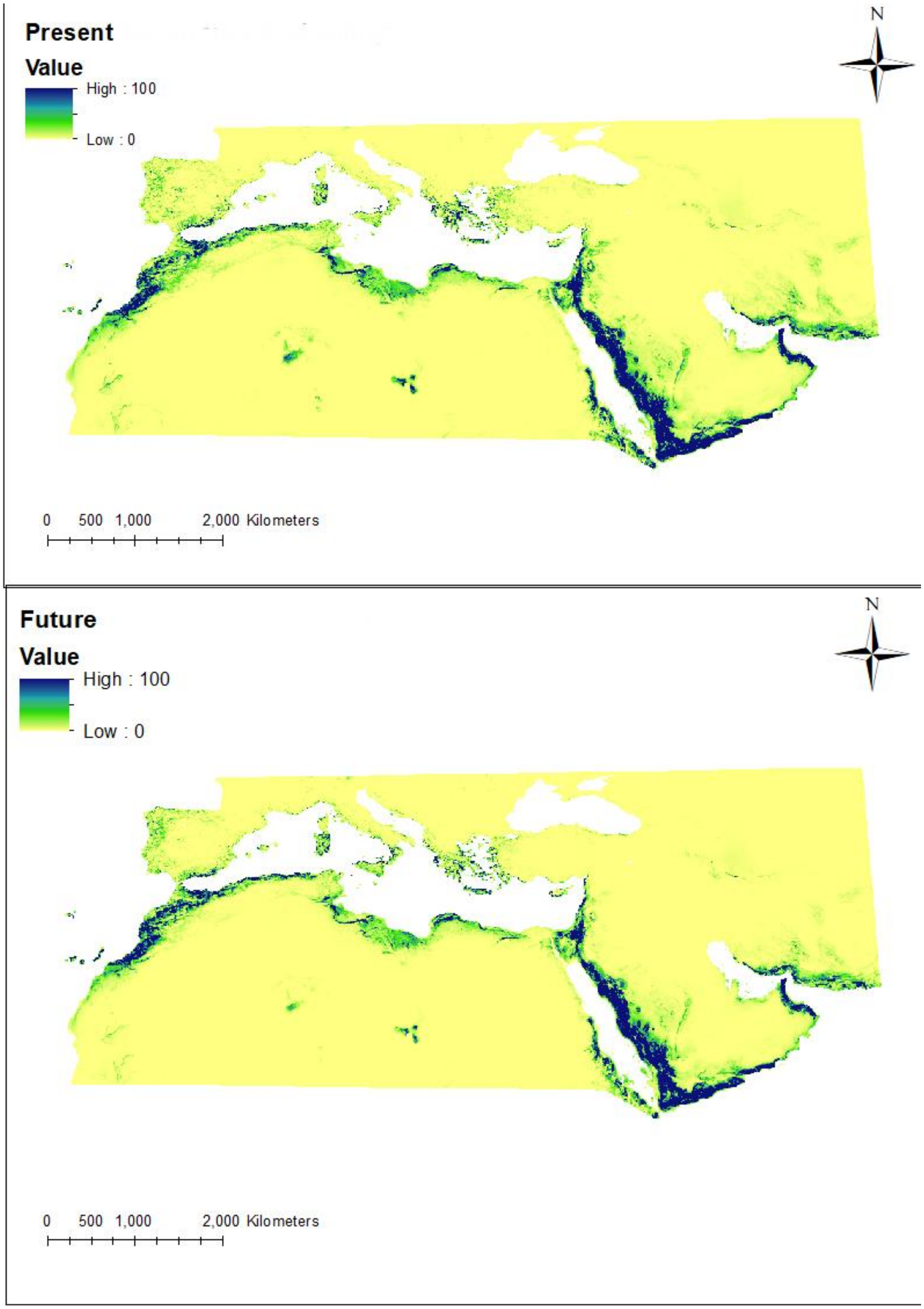

Figure S6 - Modelling output continuous maps showing predicted environmental suitability for *Panthera pardus nimr* (Arabian leopards) under present (top) and future (2050, ssp585; bottom) conditions.

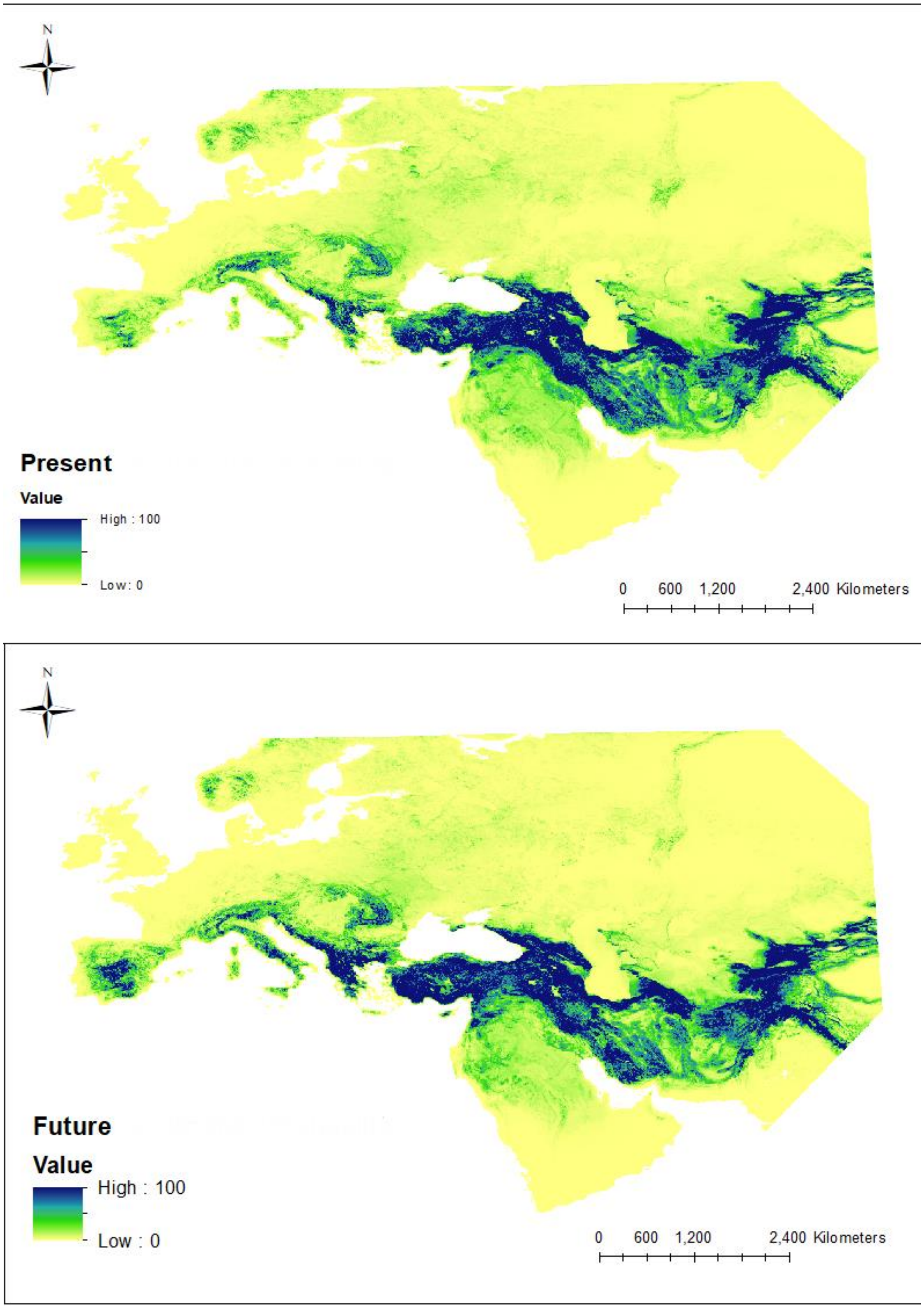

Figure S7 - Modelling output continuous maps showing predicted environmental suitability for *Panthera pardus tulliana* (Persian leopards) under present (top) and future (2050, ssp585; bottom) conditions.
